# Directed Evolution of a Yeast-Displayed HIV-1 SOSIP gp140 Spike Protein toward Improved Expression and Affinity for Conformational Antibodies

**DOI:** 10.1371/journal.pone.0117227

**Published:** 2015-02-17

**Authors:** Sebastian K. Grimm, Michael B. Battles, Margaret E. Ackerman

**Affiliations:** Thayer School of Engineering at Dartmouth, Hanover, New Hampshire, United States of America; Tulane University, UNITED STATES

## Abstract

Design of an envelope-based immunogen capable of inducing a broadly neutralizing antibody response is thought to be key to the development of a protective HIV-1 vaccine. However, the broad diversity of viral variants and a limited ability to produce native envelope have hampered such design efforts. Here we describe adaptation of the yeast display system and use of a combinatorial protein engineering approach to permit directed evolution of HIV envelope variants. Because the intrinsic instability and complexity of this trimeric glycoprotein has greatly impeded the development of immunogens that properly represent the structure of native envelope, this platform addresses an essential need for methodologies with the capacity to rapidly engineer HIV spike proteins towards improved homogeneity, stability, and presentation of neutralizing epitopes. We report for the first time the display of a designed SOSIP gp140 on yeast, and the *in vitro* evolution of derivatives with greatly improved expression and binding to conformation-dependent antibodies. These efforts represent an initial and critical step toward the ability to rapidly engineer HIV-1 envelope immunogens via directed evolution.

## Introduction

Broadly neutralizing antibodies (bnAbs) are widely thought to represent the best means of preventing HIV infection, making development of an immunogen capable of raising bnAbs a cornerstone and priority of vaccine development efforts [1,2]. Unfortunately, various factors relating both to the characteristics of known bnAbs as well as the HIV virus itself have pointed toward substantial obstacles to the realization of this goal. The sequence diversity and instability of the trimer, its heavily glycosylated structure, low surface density on the viral particle, and limited access to functionally critical epitopes have confounded efforts to induce bnAbs by vaccination [1,3]. Furthermore, cues from natural infection suggest that monoclonal bnAbs are uncommon, arise after years of infection and high viral load, fail to control established infection, must have precisely oriented binding interactions, and often have unusual properties [4–11], indicating that without a fundamental breakthrough in immunogen design, the generation of such bnAbs by vaccination is likely to remain a daunting challenge [12,13].

Even so, exciting progress has been achieved recently in characterizing the neutralizing capacity of antibodies generated in the course of natural infection [11,14–17], as well as in identifying novel bnAbs [18–27]. These and other bnAbs have greatly informed immunogen design, highlighting new regions of the envelope trimer, variable loops, envelope glycan, the membrane proximal region, novel quaternary epitopes, and receptor and co-receptor binding sites as epitopes with a combination of sufficient conservation and functional relevance to be key targets of an effective antibody response. It is anticipated that with the continued use of high-throughput B-cell screening methods, the set of bnAbs with different fine-epitope specificities and viral coverage will continue to grow and provide a rich set of probes to reinvigorate and diversify immunogen design efforts. However, a high-throughput and adaptable platform is required to ensure these findings are efficiently translated into candidate immunogen development.

In the context of natural infection, bnAbs have tended to be isolated from individuals with high viral loads, persistent antigen exposure, and progressive disease. In the absence of replicating vectors, it is difficult to envision how similar levels of antigen exposure could be accomplished via vaccination. Additionally, envelope diversity may be a key driver in the generation of neutralization breadth, posing another fundamental challenge. Together, the antigen exposure associated with natural infection likely represents both orders of magnitude greater levels and diversity than can be achieved by current strategies, leading to the discouraging conclusion that a successful immunogen may need to possess an orders of magnitude improved capacity to elicit bnAbs over natural envelope.

With these technical and immunological gaps in mind, we sought to establish a yeast surface display (YSD) platform to apply directed molecular evolution principles to the development of HIV envelope variants with fundamentally improved biophysical properties. YSD allows the display of millions of sequence variants and selection based on flexible design criteria to allow efficient and deep coverage of the envelope structure:function landscape, representing a potentially enabling technology for the rapid translation of findings from basic studies to the development of novel candidate immunogens. As such, YSD has been established as a powerful method to engineer diverse proteins for a broad range of functional improvements, including stability, specificity, affinity, catalysis, and enantioselectivity [28–31]. Routinely, variants with million-fold improvements can be isolated from large libraries, and repeated cycling of mutagenesis and selection has resulted in evolution of some of the highest affinity synthetic interactions ever observed [32]. Indeed, promising efforts aimed at the development of scaffolded epitopes and gp120 cores with desirable properties such as enhanced recognition of germline antibody families have routinely relied upon such combinatorial approaches and YSD-based directed evolution [33,34]. However, yeast expression and display of complete gp120, much less gp140, has not been described.

Here we investigated yeast as a host for the display of HIV spike protein variants and report for the first time the display of full-length gp140 on *S*. *cerevisiae*. A JR-FL gp140 variant with the SOSIP mutations served as starting point because it has previously been engineered for stability and homogeneity, was well-expressed in mammalian cells [35–37] and because the SOSIP mutations proved key to enabling structural insights into the native HIV-1 envelope trimer [38–41]. We have applied random mutagenesis and DNA shuffling in combination with stringent VRC01 driven selections to isolate gp140 SOSIP variants with significantly enhanced expression and that bind to conformational HIV bnAbs, suggesting proper antigenicity. The yeast-displayed gp140 SOSIP variants described here demonstrate the feasibility of production of HIV envelope in yeast, and the advantages of this robust platform in evolving variant envelopes. If trimeric presentation can be confirmed or further engineered, YSD may prove a robust platform for the rapid evolution and selection of promising vaccine immunogens.

## Results

### Influence of N-glycosylation on binding of conformational HIV antibodies to yeast-produced gp120

Since HIV spike proteins are highly glycosylated, proteolytically processed, and contain numerous disulfide bonds, only eukaryotic cells with appropriate protein processing machinery can serve as hosts for the display of functional protein. Whereas mammalian cell lines are relatively slow growing, and achieving the single genotype-phenotype linkage requires viral transfections or other rather cumbersome and time-consuming methodologies, the yeast *S*. *cerevisiae* is a robust and well-described eukaryotic host for cell-surface display [42]. Yeast are capable of displaying complex mammalian glycoproteins such as antibody fragments, full-length antibodies, peptide-MHC molecules, or growth factor receptors [42–45], as well as a growing number of viral envelope proteins of fragments thereof such as hemagglutinin, the gp120 core, Dengue E protein, SARS-CoV, West Nile Virus envelope, and the Hepatitis C Virus envelope protein E2 [46–51], suggesting that the display of HIV envelope protein is feasible in principle. Nevertheless, in *S*. *cerevisiae*, N-linked glycans are extended and mannose-rich [52], though *P*. *pastoris* yeast strains with human or human-like N-linked glycosyation have been reported to overcome this limitation [53,54].

To investigate whether heterologous N-glycosylation in yeast has a major effect on the recognition of gp120 by conformational antibodies, we compared HIV-1 JR-FL gp120 produced in either *S*. *cerevisiae* strain YVH10 or glycan-engineered *P*. *pastoris* [53] for binding to a panel of conformational HIV-1 bnAbs. The mannose-rich N-glycosylation in YVH10 is trimmed to a more human-like 5-mannose stem in engineered *Pichia*, which may promote proper protein folding and conformational Ab recognition. However, we found that *Pichia*-produced gp120 did not display a clearly improved binding profile across a panel of bnAbs (S1 Fig.). Furthermore, *P*. *pastoris*-produced gp120 appeared somewhat more fragmented on a Western blot, suggesting more proteolytic degradation as compared to *S*. *cerevisiae*. Therefore, and because of the well-established and characterized Aga1/2-based display system, *S*. *cerevisiae* was chosen as host for HIV spike protein display.

### Design of a codon-optimized gp140 for expression in *S*. *cerevisiae*

**Fig 1:**
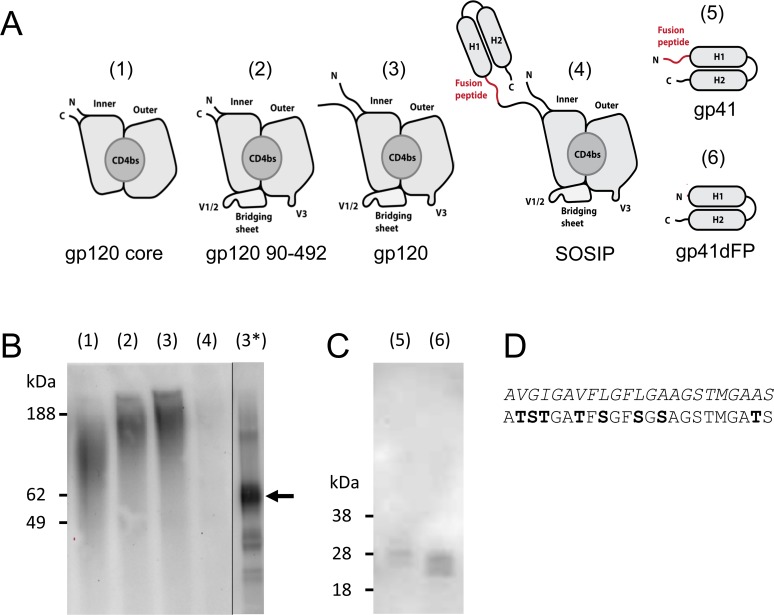
Expression analysis of different gp140 fragments. (A) Schematic representations of expressed HIV-1 gp140 fragments, including gp120 inner and outer domain (inner and outer), CD4 binding site (CD4bs), V1/2 loop region (V1/2), the V3 loop (V3) and helical regions 1 and 2 of gp41 (H1, H2). (B) Western blot analysis of (1) yeast-secreted YU2 gp120 core, (2) gp120 residues 90–492, (3) gp120, (4) JR-FL SOSIP gp140, (3*) PNGaseF-treated YU2 gp120 with marked main band. Lane (3*) is non-adjacent but originates from the same blot. (C) Western blot analysis of (5) yeast-secreted JR-FL gp41 and (6) gp41 lacking the fusion peptide. (D) Amino acid sequences of the original and the modified fusion peptide, with modified residues in bold text.

Since the secretion of a full-length JR-FL gp140 including the stabilizing SOS and I559P substitutions (SOSIP JR-FL gp140 [36,37]) in *S*. *cerevisiae* YVH10 had failed, we fragmented the gene and compared the level of secreted protein between a gp120 stripped core construct described previously [47], gp120 trimmed at N- and C-termini, full-length gp120, a full-length SOSIP gp140, as well as gp41, and gp41 lacking its hydrophobic fusion peptide (Fig. 1A). Notably, proteins were not secreted as Aga2 fusions in this study. Firstly, both gp120 fragments and full-length gp120 could be secreted at clearly detectable levels, but not full-length SOSIP gp140. Secondly, the omission of the fusion peptide clearly enhanced secretion of gp41, suggesting that its omission or modification may also improve full-length gp140 secretion. Thirdly, gp120 collapsed near its expected MW of 52 kDa after treatment with N-glycosidase PNGase F, suggesting extensive N-glycosylation (Fig. 1B and 1C). Based on these findings, we rationally designed a JR-FL SOSIP-based gp140 (designed, or d-SOSIP) with some modifications: codons were optimized for *S*. *cerevisiae* [55]; hydrophobic amino acids within the fusion peptide were replaced by structurally related but more hydrophilic residues, which if possible were found among circulating viral sequences (Fig. 1D); and lastly the Furin protease cleavage site was substituted with a yeast Kex2 recognition site, and a predicted internal Kex2 site was removed. We then compared secreted protein content in cell culture supernatant and found that d-SOSIP, but not the initial JR-FL SOSIP gp140 could be detected at significant levels. The d-SOSIP variant was significantly N-glycosylated, and collapsed after PNGase F-treatment (S1C Fig.).

### Characterization of d-SOSIP displayed on *S*. *cerevisiae*

**Fig 2:**
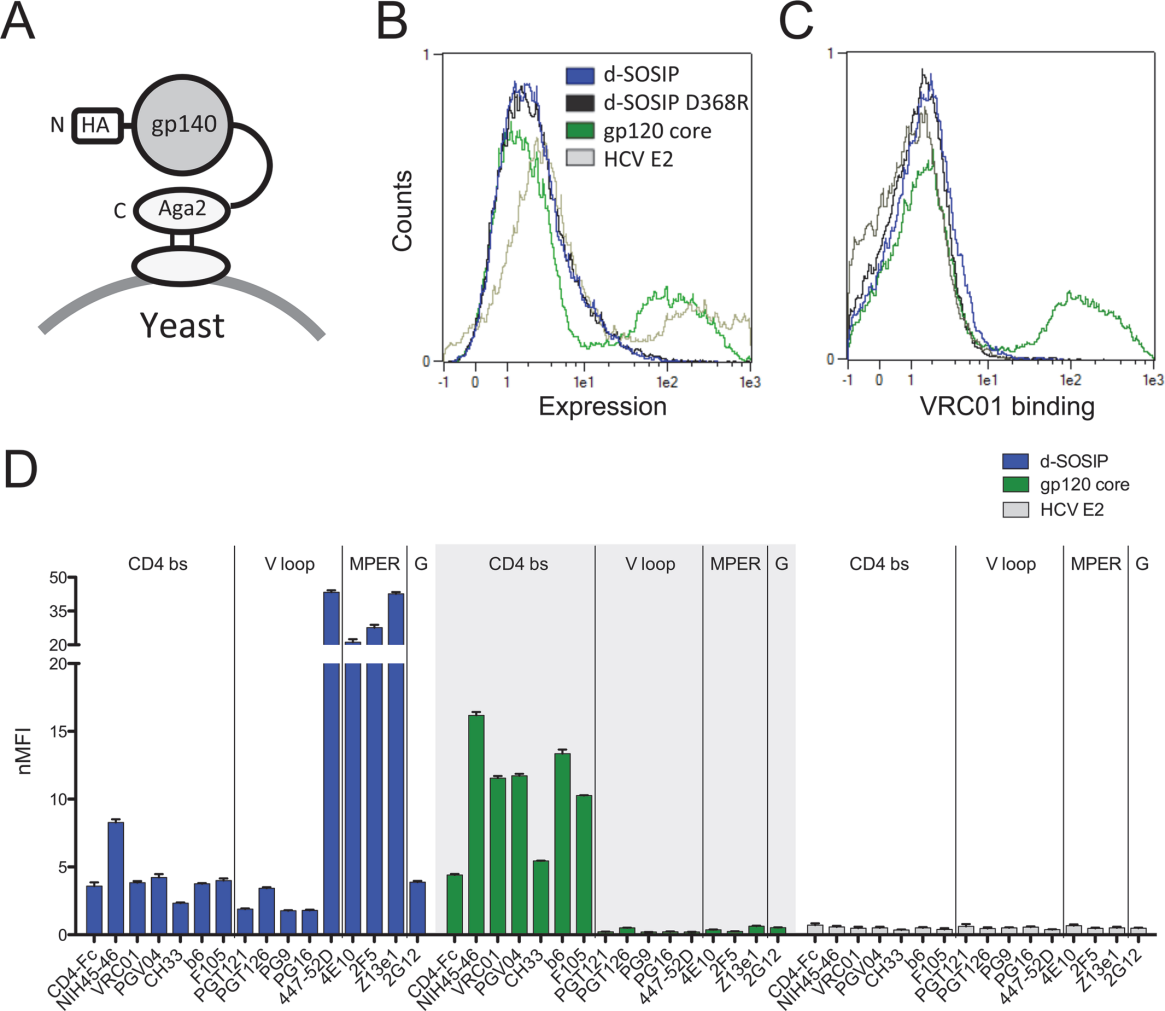
Binding analysis of yeast-displayed spike protein variants. (A) Schematic illustration of yeast-displayed gp140 that is C-terminally fused to Aga2. (B) Overlay of histograms obtained from *S*. *cerevisiae* displaying JR-FL d-SOSIP gp140 (d-SOSIP), JR-FL d-SOSIP gp140 D368R mutant (d-SOSIP D368R), YU2 gp120 core (gp120 core) or an Hepatitis C Virus reference protein (HCV E2) probed for display level. Counts are plotted against fluorescence on logarithmic scale. (C) Overlay of histograms of yeast-displayed constructs binding to 100 nM HIV bnAb VRC01. (D) Median fluorescence intensities (MFI) normalized for yeast display levels (nMFI) are plotted for a panel of HIV Abs, grouped by epitope (CD4bs = CD4 binding site; V loop = variable loops V1/2/3; MPER = membrane proximal extracellular region; and G = glycan-specific). Error bars represent standard deviations of triplicate measurements.

The rationally designed d-SOSIP variant and a mutant with disrupted CD4 binding site (CD4bs)-specific Ab binding (d-SOSIP D368R) were displayed as Aga2 fusion proteins on yeast (Fig. 2A), and compared for display level and binding to a panel of HIV bnAbs together with the well-characterized and folded YU2 gp120 core [47] and an unrelated viral envelope protein (E2) derived from Hepatitis C virus (HCV E2) as positive and negative controls (Fig. 2). Notably, gp120 core epitopes are included in d-SOSIP, while the gp120 core lacks many of the epitopes of d-SOSIP, including variable loop and gp41-associated epitopes (Fig. 1A). Induced yeast cells always included a population that did not display the heterologous protein, which is a feature of the Aga2 display system [42]. The d-SOSIP constructs showed extremely low display levels as compared to the gp120 core (Fig. 2B), and only somewhat above background binding of the CD4bs-specific VRC01 Ab (Fig. 2C). However, standardizing the binding of HIV-specific Abs when variable display level was taken into account lead to detectable binding across a wide range of Ab specificities, including conformational, linear, and glycan-dependent sites. Yeast-displayed d-SOSIP bound well to the V3-specific Ab 447–52D and to MPER Abs, as might be expected due to their ability to bind to unconstrained peptides. Surprisingly, low but reproducible signal was observed, suggestive of recognition by glycan and trimer-preferring antibodies such as PGT121/126 [56] and PG9/16 [24] (Fig. 2D and S2 Table). Furthermore, the d-SOSIP D368R substitution was observed to reduce binding of CD4bs antibodies. Nonetheless, the low signal relative to gp120 core for many of the antibodies tested clearly indicated significant room for improvement, which might be achieved by coupling the rational design modifications made thus far with directed evolution strategies.

### Selection of gp140 variants with improved binding to conformational mAbs

**Fig 3:**
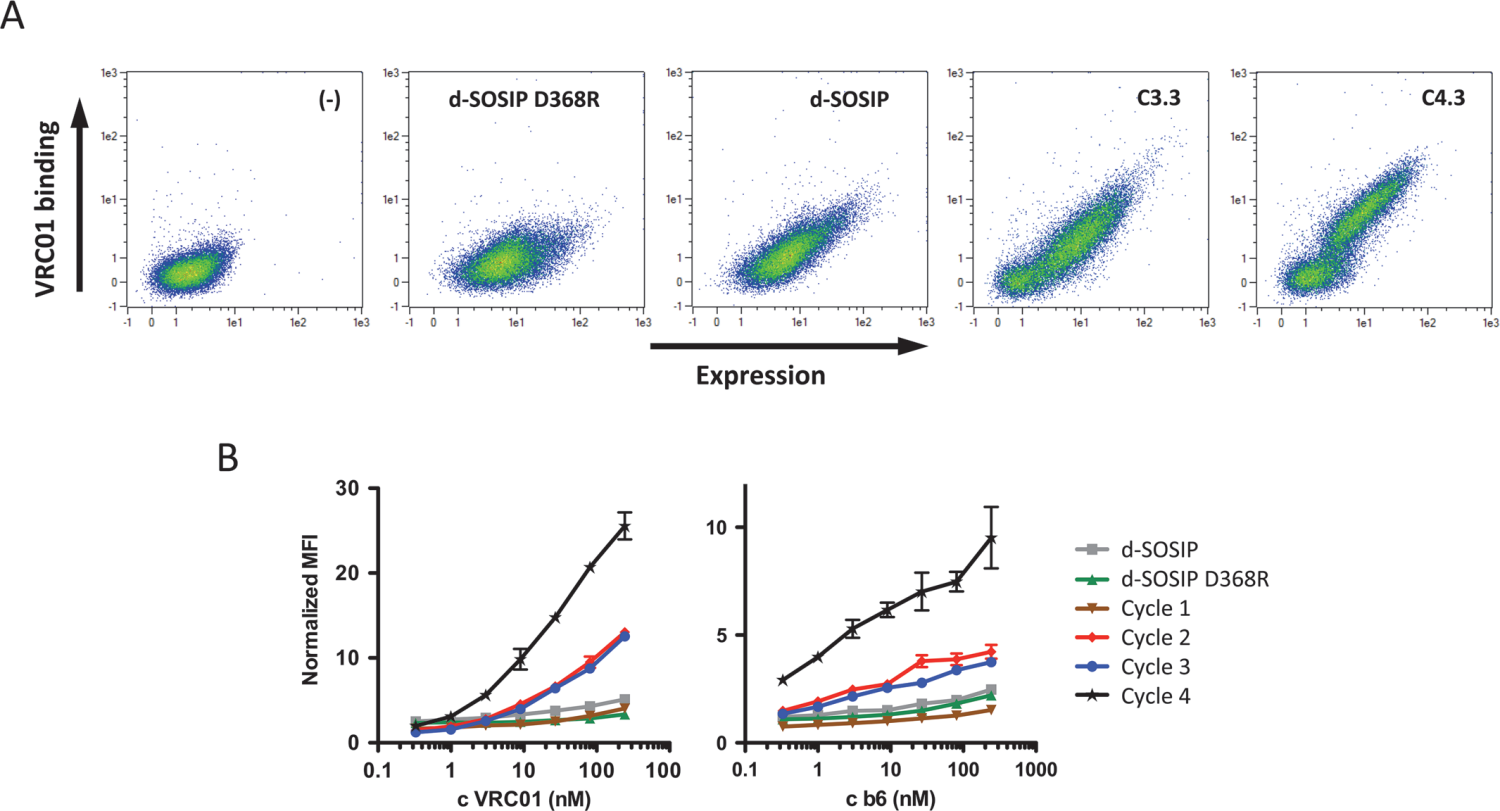
Pool binding analysis of selection rounds. (A) Scatter plots obtained from 20,000 yeast cells displaying no HIV envelope protein (-), d-SOSIP gp140 D368R (d-SOSIP D368R), d-SOSIP or selected pools after cycles C3.3 and C4.3, respectively, probed for binding to 27 nM VRC01. (B) Binding isotherms obtained from pools selected after round 1.3, 2.4, 3.3 and 4.3 (cycle 1, 2, 3, 4), and reference clones d-SOSIP gp140 (d-SOSIP) and d-SOSIP D368R to HIV mAbs VRC01 or b6. Expression-normalized median fluorescence intensity (nMFI) is plotted against antibody concentration. Error bars represent standard deviations of triplicate measurements.

To select for properly folded sequence variants of d-SOSIP, a library of 2.0x10^7^ members was prepared, with each library member containing an average of about 6.7 random amino acid mutations (Table 1). Conformation-dependent mAbs that specifically bind to discontinuous epitopes served as molecular probes for properly folded antigens. Here, the d-SOSIP library was extensively screened for improved binding to the conformation-dependent CD4bs bnAb VRC01, which mimics the natural ligand CD4 in its mode of binding[57]. The pool of clones selected was further diversified to up to 7.7x10^7^ members during two additional cycles of error prone PCR and repeated FACS selection for improved binding to either VRC01 (track 1), alternating enrichment based on binding to HIV CD4bs Abs VRC01, CH31 or PGV04 (track 2), or against a pooled mix of the five CD4bs mAbs VRC01, PGV04, CH31, b12 and b6 (track 3), as described in Table 1. These mAbs were chosen because they all bind to the conformational, conserved and functionally critical CD4bs, while being different in sequence and paratope, so as to minimize the risk of selecting specific affinity-enhancing contact residues instead of general structurally stabilizing mutations. A comparative binding analysis of the initial d-SOSIP and the selected pools from cycle 2 onward showed enhanced binding not only to target bnAb VRC01, but also to non-target CD4bs mAb b6, suggesting a generally improved structural representation of the conformational CD4bs epitope (Fig. 3B), as VRC01 and b6 significantly differ in their epitope footprints within the CD4bs [58,59]. DNA sequencing revealed that two dominating and distinct clusters of sequences had been enriched (S3A Fig.). To select for combinatorial mutants with further improved binding properties among those clusters, and to remove structurally disruptive mutations that likely had been co-selected, d-SOSIP and the 3.3 pool variants were DNA-shuffled [60] at a 1:1 ratio, and then subjected to an additional three rounds of FACS following selection tracks 1, 2 and 3. The selected 4.3 population pools showed both greatly enhanced expression and binding to CD4bs mAbs VRC01 and b6 as compared to the 3.3 pool selected before DNA shuffling (Fig. 3), while there was no difference in binding observed between tracks 1, 2 and 3 (S4 Fig.).

**Table 1:** Summary of library size, diversity, and selection parameters and paths.

Cycle	*Method*	*Size*	*Mutations*	Track (1)	Track (2)	Track (3)	Counter selection
1.1	epPCR, FACS	2.0x10^7	6.7 aa/gene	256 nM VRC01	-	-	No
1.2	FACS	-	-	256 nM VRC01	-	-	No
1.3	FACS	-	-	128 nM VRC01	-	-	No
2.1	epPCR, FACS	5.3x10^7	12.3 aa/gene	256 nM VRC01	-	-	No
2.2	FACS	-	-	128 nM VRC01	-	-	No
2.3	FACS	-	-	128 nM VRC01	-	-	No
2.4	FACS	-	-	128 nM VRC01	-	-	Yes
3.1	epPCR, FACS	7.7x10^7	19.0 aa/gene	128 nM VRC01	-	-	No
3.2	FACS	-	-	64 nM VRC01	64 nM PGV04	64 nM Ab mix	No
3.3	FACS	-	-	32 nM VRC01	64 nM CH31	32 nM Ab mix	No
4.1	Shuffling, FACS	6.2x10^7	6.1 aa/gene	32 nM VRC01	32 nM VRC01	32 nM VRC01	No
4.2	FACS	-	-	16 nM VRC01	32 nM CH31	16 nM Ab mix	No
4.3	FACS	-	-	2 nM VRC01	8 nM PGV04	4 nM Ab mix	No

### Characterization of selected clones

**Fig 4:**
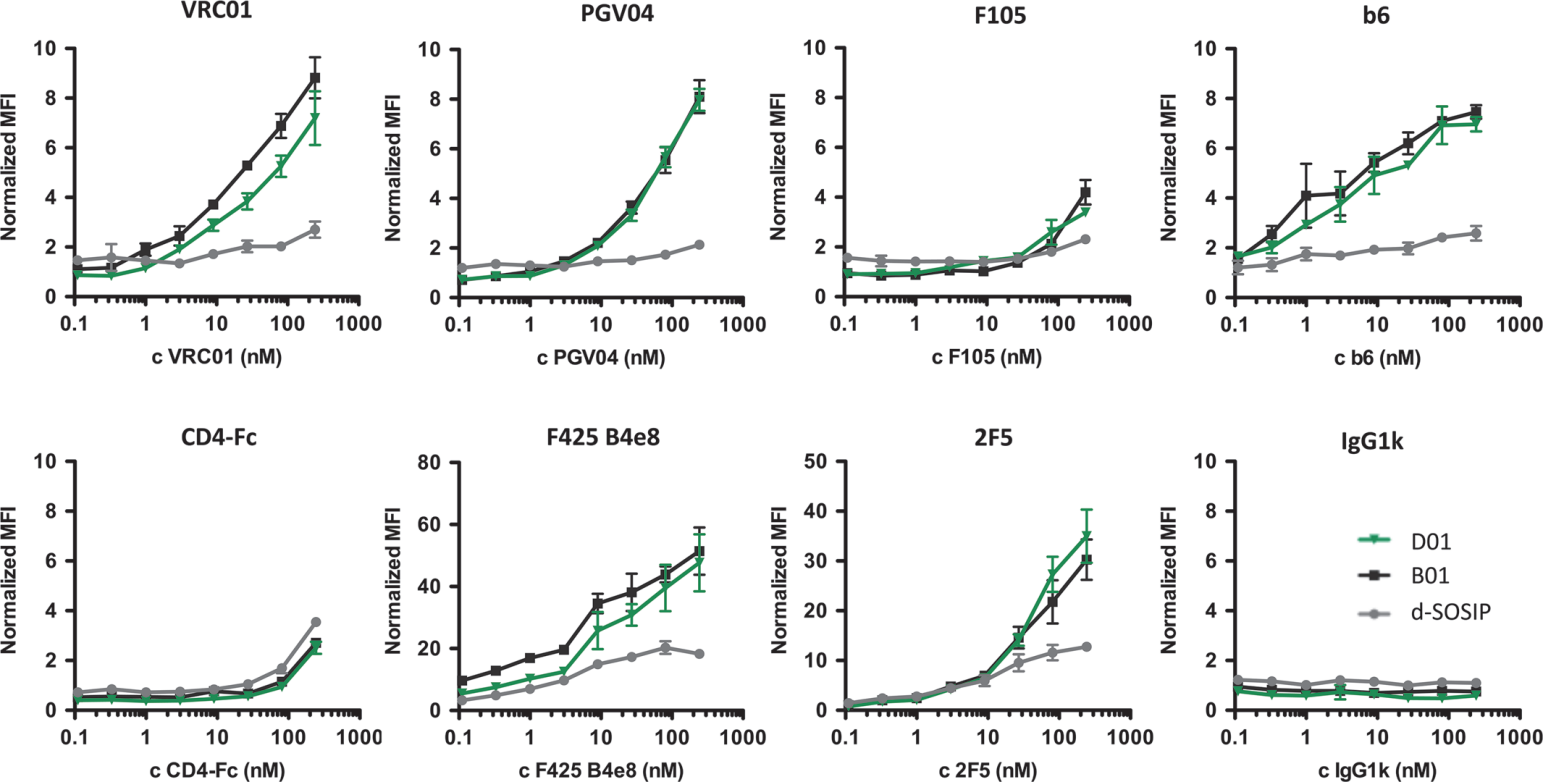
Single clone binding analysis. Expression-normalized median fluorescence intensity (MFI) is plotted against logarithmic Ab concentrations for d-SOSIP (gray) and clones 4.3.B01 (black) and 4.3.D01 (green) selected after DNA shuffling. Binding isotherms to a human IgG1k control Ab are included as reference. Error bars represent standard deviations of triplicate measurements.

A total 16 clones from each selection track were evaluated for binding to VRC01 and absence of binding to two negative control mAbs. Using an aggregated ranking analysis, the 12 clones with the best VRC01 and least control mAb binding were further screened for broad recognition of conformational CD4bs mAbs PGV04 and b6, as well as the natural ligand CD4 and gp41 membrane-proximal extracellular region mAb 2F5. Two unique clones, denoted 4.3.B01 and 4.3.D01, were identified as ranking best both for binding specificity and breath of HIV-specific mAb/CD4 recognition. DNA sequencing of 4.3.B01, 4.3.D01 and a reference clone that was enriched before DNA shuffling (3.3.1) revealed a total of 17, 12 and 9 amino acid substitutions in 3.3.1, 4.3.B01, and 4.3.D01, respectively. Notably, 4.3.B01 and 4.3.D01 are identical in the gp120 region and only differ by 3 mutations within the gp41 region that were reverted to d-SOSIP wild type for clone 4.3.D01 (S5 Fig.). Both 4.3.B01 and 4.3.D01 showed greatly improved binding to several CD4bs Abs, both neutralizing and non-neutralizing, and also V3 Ab F425 B4e8 and MPER Ab 2F5, as compared to the initial d-SOSIP. Perhaps surprisingly, improved binding to natural ligand CD4-Fc was not detected, but binding was maintained at a comparable level with d-SOSIP (Fig. 4).

To determine whether the enhancements observed in displayed variants were generalizable to secreted protein, the clone 4.3.D01 was expressed solubly. The evolved variant was subsequently compared to yeast-secreted gp120 for binding to envelope-specific antibodies. Notably, reference d-SOSIP could not be secreted at sufficient quantities in yeast for binding analyses, in line with the extremely low display level of d-SOSIP observed on yeast cells (Figs. 2 and 3). Beyond enabling expression, the 4.3.D01 clone exhibited improved binding to a number of mAbs, notably NIH45–46 G54W, relative to gp120. (S7 Fig.), suggesting that the improved binding properties observed on yeast cells correlate with improved binding properties of soluble gp140.

### Mapping selected mutations onto the structure of BG505 SOSIP.664

**Fig 5:**
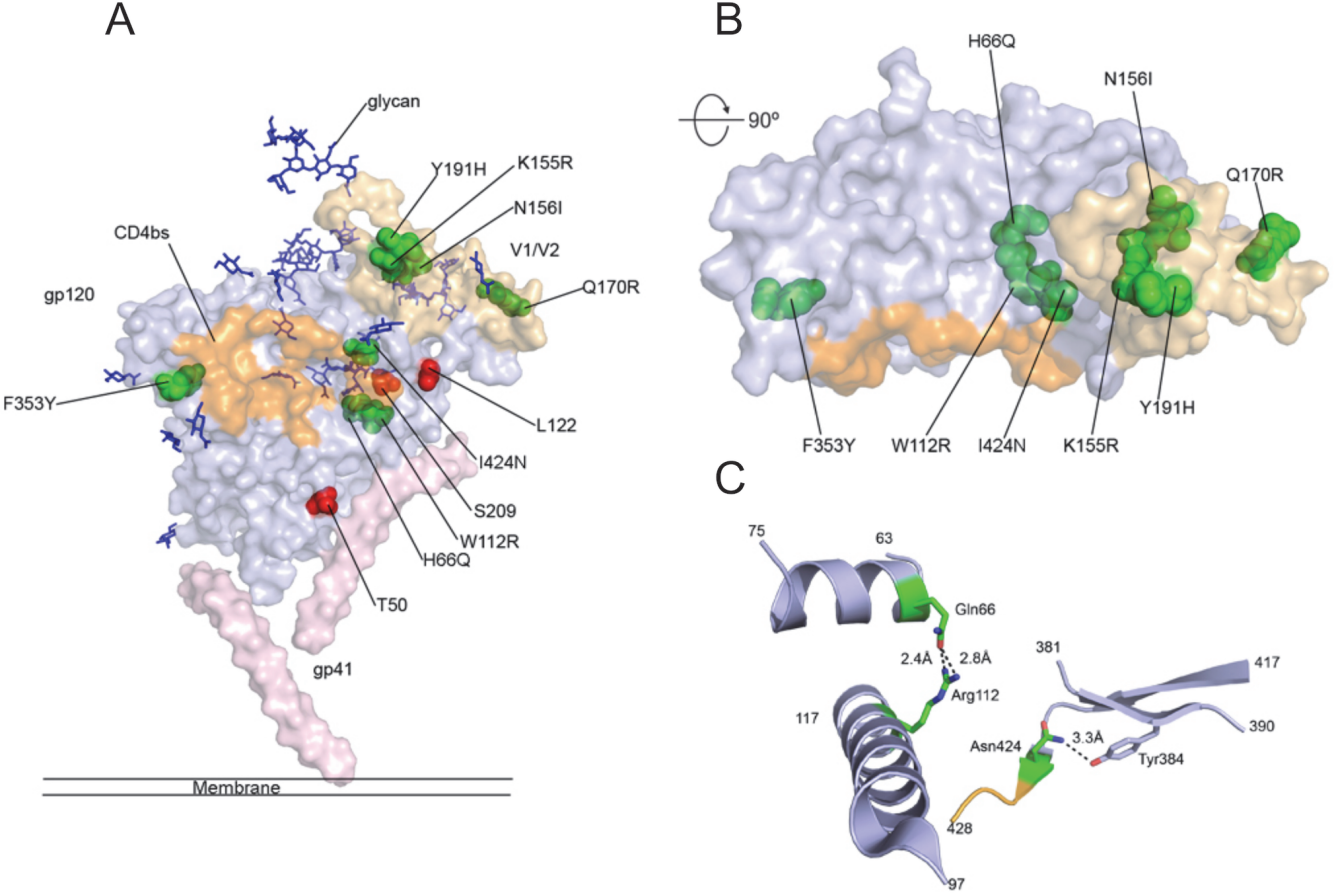
Mapping of mutations on gp140 structure. (A) Surface view of the structure of a BG505 SOSIP.664 gp140 monomer. The gp120 unit is shown in light blue and the gp41 unit in light pink. Glycans are shown as blue sticks, the CD4bs is orange and the V1/2 loop domain golden. Mutated residues found in clone 3.3.1 only are shown in red and residues found in clones 4.3.B01 and 4.3.D01 are shown in green. (B) The molecule was rotated 90° around the x-axis and residues found in 4.3.B01 and 4.3.D01 are shown in green. (C) Cartoon view of mutated residues found within gp120, putative hydrogen bonds are denoted as dashed lines.

Mapping of the mutations on the crystal structure of BG505 SOSIP.664 gp140[39] showed that the mutations found in 4.3.B01 and 4.3.D01 cluster in a relatively narrow region within the V1/V2 loop region of gp120, but proximal to the CD4bs (Fig. 5A). Their side chains are buried within the protein or located in small cavities and do not appear to directly contact CD4bs mAbs (Fig. 5B), suggesting that structurally stabilizing mutations have been selected. Interestingly, mutated residues H66Q and W112R may form hydrogen bonds stabilizing helices within the inner domain of gp120. Similarly, I424N may form a novel hydrogen bond with Y384 (Fig. 5C). Mutation N156I disrupts an N-glycosylation motif located within the V1/V2 loop region. Three additional gp120 mutations that were lost after shuffling (T50A, L122F, and S209T) were all surface located, suggesting that surface-located mutations may not be essential to improved protein folding in yeast. The mutations found within gp41 could not be mapped due to disordered electron density in this particular region of the SOSIP crystal structure. Since 4.3.B01 and 4.3.D01 showed virtually identical binding profiles and expression levels (Fig. 4), these gp41 mutations do not appear to strongly contribute to either improved protein expression or Ab recognition.

## Discussion

A key attribute of most effective anti-viral vaccine responses is development of antibodies to viral envelope proteins which result in the ability to prevent or clear viral infection [61]. To this end, induction of neutralizing antibodies capable of blocking viral infection has been a cornerstone of HIV vaccine efforts. However, HIV has an arsenal of ways to evade antibody recognition, including multiple conformational states such as closed and open, cleaved or uncleaved trimer and various monomeric spike protein forms, which are thought to distract and dilute the adaptive immune response to the infectious “native” envelope trimer [11,62–64].

Immunogen design efforts aimed to circumvent these barriers stem back to the discovery of the HIV bnAb b12, and began with the identification of epitope mimetic peptides that were capable of binding to b12 with high affinity. However, vaccination with mimetic peptides failed to induce Abs that bound to gp120 [65]. Other more recent approaches have been based on a subtractive principle, in which the immunogenic features of the antigen which lead to non-neutralizing but immunodominant antibody responses were removed or masked with glycans [58,66,67], or even more dramatically, on the direct grafting of functionally relevant sites onto alternative scaffolds [68–70], with the goal of focusing the immune response on functionally relevant epitopes. The subtraction of alternative epitopes, however, may not actively promote a response against the desired epitope, and may rather result in a blunted immune response, in which the titer of Ab recognizing the selected epitope is not enhanced, but the total antibody response is decreased. While mixed success has recently been reported from these efforts in the setting of HIV [71], the subtraction of epitopes may unnecessarily constrain the immune response—strongly limiting potentially protective recalled T cell responses, and non-productively decreasing the polyclonality of the antibody response, resulting in reduced recruitment of effector mechanisms reliant on avid interactions with antibody opsonized cells or virus. Because these effector mechanisms have a demonstrated importance in preventing acquisition, decreasing viral burden, and delaying progression [72], the possible immunologic outcome of vaccines with reduced epitopic coverage may be dividing rather than conquering.

Extensive studies utilizing soluble gp120 have generally failed to provide protective Ab responses, and numerous full-length and trimeric gp140-based antigens that may better resemble the structure of the native spike have also been tested [73–75], following the rationale that any antibody recognizing the native, trimeric spike should neutralize virus [41]. SOSIP trimers fall into this class, and a growing number of envelope sequences with SOS and IP mutations have been individually constructed and tested, culminating with the BG505 SOSIP.664 gp140 trimer [39].

A complementary approach to the strictly rational development of stable, uniform and well-behaved proteins is combinatorial protein engineering, involving screening of large libraries of protein mutants displayed on viruses or cells for improved properties such as affinity, stability or protein expression. Toward this end, HIV virus particles have been exposed to temperature or chemical denaturant stress and screened for retained infectivity of mammalian cells. More homogeneous and stable viruses could be selected following this procedure [76]. However, cellular protein display methods, yeast display in particular, have standing as the gold-standard for evolution of complex and glycosylated proteins based on advantages in speed, library construction, scalable selections, and ease of use.

Our application of this system to a designed variant of JR-FL SOSIP gp140 represents the first use of a high-throughput platform for whole envelope evolution. The ease with which variants with improved expression and binding to conformational HIV antibodies could be evolved from repeated rounds of random mutagenesis and flow-cytometric selection points toward promising utility. While the mannose-rich glycosylation of *S*. *cerevesiae* in theory reduces enthusiasm about this host, major classes of bnAbs, including glycan-specific Abs, bound to yeast-produced gp140 variants, and loss of only one N-glycosylation motif (N156I) was observed after selection. While improved binding to CD4 was not observed, the selected variants exhibited similar CD4 binding as gp120 core, and showed broadly improved binding across multiple CD4bs mAbs, suggesting a generally improved structural representation of this epitope.

Whether the selected d-SOSIP variants could elicit improved neutralizing immunity when applied as immunogens is unclear. Because improved antigenicity does not equate to improved immunogenicity, immunization studies would be required to evaluate their ability to elicit neutralizing antibodies. Differences in glycosylation can also have a large impact on antigenicity [17], a factor that has not been optimized in our study. Importantly, while yeast utilize the same glycosylation motif, the composition of the glycan incorporated differs significantly, and could be engineered. We rather consider the methodology described here and gp140 variants as an enabling platform for future investigations towards development of improved immunogens. A panel of diverse, stabilized gp140 variants is thought to likely be required to cover circulating virus diversity, minimize immune escape by mutation and glycan masking, and elicit broad neutralization activity [15].

The nine mutations within gp120 that contributed to the enhanced properties in yeast are located in a relatively narrow region, either in proximity of the CD4bs or within the V1/V2 loop domain, as determined by homology modeling. Interestingly, three of the nine mutations (H66Q, W112R and I424N) may form novel hydrogen bonds. The predominantly buried locations of the mutations identified and the predicted presence of additional, buried hydrogen bonds in selected clones suggest that the poor surface display level and limited binding of yeast-displayed gp140 to conformational Abs observed initially were related to poor folding of gp140 in *S*. *cerevisiae*, and that improved gp120 core packing and hydrogen bonding within the core may have helped to stabilize the protein, enhancing folding and resulting in higher display levels. In fact, the display level of proteins fused to Aga2p was shown previously to correlate with their thermal stability and soluble expression levels [77], and yeast is known to contain a chaperone machinery that can retain and degrade misfolded protein [78]. Further, the accumulation of mutations within the V1/V2 loop domain suggest that this region, in addition to the gp120 core itself, had a critical impact on obstructing proper folding of gp120. This finding is further supported by the notion that yeast produced gp120 core lacking this domain binds well to CD4bs conformational Abs, while full-length gp120 containing V1/V2 did not. The more hydrophilic gp41 fusion peptide has provided a critical handle to rationally improve expression of d-SOSIP gp140 in *S*. *cerevisiae*, however no additional expression enhancing or apparently structurally stabilizing gp41 mutations were identified in the evolved d-SOSIP gp140 variants. Evidence as to the utility of these rational mutations can be found in the absence of reversions observed among selected clones.

Notably, this study does not provide any evidence for the display of gp140 trimers on yeast, which from a steric perspective seems unlikely due to clashes of the Aga2 cell wall anchoring fusion partners. Further, the engineering of the fusion peptide towards reduced hydrophobicity may, while being as conservative as possible, have an effect on an overall trimer structure. To address this, in future studies, a secretion-capture-based display system could be developed [79], allowing for the evolution of trimeric gp140 spike protein libraries that are non-covalently linked to Aga2. The engineered fusion peptide could gradually be mutated back to wild-type if it affects trimer structure, or fusion peptide variants could be selected from yeast-displayed libraries that improve trimer-specific antibody recognition.

In summary, the d-SOSIP gp140 variants evolved and the platform and approach described here provide a step forward toward the development of promising novel HIV immunogens, which could be rapidly and iteratively evaluated in immunization studies in animal models. Such evaluation could help elucidate the value of whole envelope strategies relative to subtractive approaches focusing on a restricted set of epitopes. A less hydrophobic fusion peptide proved to be a critical feature to achieve an initial detectable expression level allowing for yeast display-based cell sorting. The shuffling of accumulated mutations and backcrossing to wild type greatly boosted binding to the target HIV mAbs and potentially eliminated deleterious mutations. Variants with further improved binding to certain HIV bnAbs or their germline precursors, following the concept of B-cell-lineage immunogen design [80,81] may be evolved relatively quickly using the approach described here. Glyco-engineered yeast strains [82] could serve as hosts for future yeast-based display systems to select spike protein variants homogeneously decorated with human-like glycan that is involved in binding of many bnAb-classes such as pg9/16[24] or pgt121–123 [56]. Such evolved yeast-produced spike protein derivatives may also be well suited for solving additional spike protein structures, facilitating rational vaccine design approaches, and further contributing to the immunogen pipeline.

## Materials and Methods

### General

PCR products were prepared using proofreading Phusion DNA polymerase (Finnzymes) and oligonucleotide primers from Integrated DNA Technologies, unless otherwise stated. DNA restriction and modifying enzymes were from New England Biolabs. PCR products were purified and gel-extracted using kits from either Qiagen or Geneaid. DNA sequences of all constructs were verified using an Applied Biosystems Model 3100 DNA sequencer. *E*. *coli* strain DH5 alpha was used as host for molecular cloning. Yeast strains *S*. *cerevisiae* YVH10 or *P*. *pastoris* SuperMan5 (Research Corporation Technologies) were used as hosts for soluble protein secretion and *S*. *cerevisiae* EBY100 for cell surface display.

### Preparation of DNA constructs

Plasmids pBGP1-JRFL-gp120, pBGP1-JRFL-gp41 and pBGP1-JRFL-gp41dFP for soluble protein secretion in *P*. *pastoris* strains were prepared as follows. HIV-1 JR-FL gp120, gp41 and gp41dFP were amplified from pPPI4-JRFL-SOSIPopt_R6 gp140[36] using primers JRFL-for2 and JRFLgp120-rev2, JRFLgp41-for2 and JRFL-rev3 or JRFLgp41dFP-for and JRFL-rev3 and inserted between the EcoRI and XbaI sites of pBGP1[83]. Plasmids pRS-JRFL-gp140, pRS-JRFL-gp120H and pRS-JRFL-gp120 for soluble protein secretion in *S*. *cerevisiae* YVH10 were prepared by amplification of gp140, gp120H or gp120 using primers JRFL-for and JRFL-rev2, JRFL-for and JRFLgp120H-rev or JRFL-for and JRFLgp120-rev and insertion between EagI and BamHI sites of pRS-4G[47]. For plasmid pRS-d-SOSIP, a yeast codon-optimized and designed HIV-1 JRFL gp140, denoted d-SOSIP, based on JRFL-SOSIPopt_R6 gp140[36] was constructed (DNA2.0) and inserted between EagI and BamHI sites of pRS-4G. The DNA sequence of d-SOSIP is shown in S6 Fig. Plasmid pCHA was obtained as a kind gift from Jordi Mata-Fink [47]. Plasmids pCHA-d-SOSIP and pCHA-JRFL-gp120 for *S*. *cerevisiae* cell surface display were prepared by amplification of gp140 or gp120 from pRS-d-SOSIP using primers gp120co-for and gp140co-rev or gp120co-rev and insertion of PCR products between NheI and BamHI sites of pCHA, resulting in an orientation of HA tag, d-SOSIP, c-myc tag, followed by Aga2p from the N to C termini, respectively. pRS-d-SOSIP-D368R was prepared using primers JRFLD368R-for and JRFLD368R-rev, following the Quikchange site-directed mutagenesis procedure (Agilent). PCR primers are listed in S1 Table.

### Protein production and purification

For secretion of soluble HIV spike protein variants lacking any cell-wall anchoring domain from *P*. *pastoris* SuperMan5, electro-competent yeast cells were prepared as described previously[84], transformed with pBGP1 constructs, plated on YPD plates containing 100 μg/l Zeocin (Invivo Gen) and incubated at 30° C for 3 days to obtain single clones. Typically 2 to 200 ml YPD cultures containing 100 μg/l Zeocin were inoculated to an OD600 of 0.1 from over-night pre-cultures and grown for 6 h at 30° C followed by 5 days at 20° C for constitutive protein expression. For secretion of soluble HIV spike protein variants lacking any cell-wall anchoring domain from *S*. *cerevisiae* YVH10, chemically competent yeast cells were prepared and transformed with pRS constructs using the Frozen-EZ Yeast Transformation II kit (Zymo), plated on SD-CAA plates supplemented with 40 mg/L L-Tryptophan and incubated at 30° C for 3 days to obtain single clones. Typically, 2 to 50 ml SDCAA cultures supplemented with 40 mg/L L-Tryptophan were inoculated from over-night pre-cultures to an OD600 of 0.2 and grown for 6 h at 30° C. For induction of protein production, cells were spun down, suspended in 200 ml SGCAA medium containing 40 mg/L L-Tryptophan and grown for 5 days at 20° C. Media compositions have been described previously[84]. Secreted spike protein variants were either detected directly in culture supernatant or IMAC-purified and buffer-exchanged to PBS prior to further analysis.

### Yeast cell surface display and flow-cytometric analysis

HIV spike protein variants were displayed on S. cerevisiae EBY100 as decribed previously[47]. Briefly, *S*. *cerevisiae* was transformed with pCHA constructs using the Frozen-EZ Yeast Transformation II kit (Zymo) and clones were grown on SDCAA plates at 30° C. Typically 2 ml SDCAA medium was inoculated to an OD600 of 0.2 from an over-night pre-culture, grown at 30° C for 6 h, transferred to SGCAA medium and grown for 12 to 18 h at 30° C. Induced cells were analyzed for cell surface display on a MACSQuant Analyzer (Miltenyi Biotec). Typically 10^7^ yeast cells were suspended in 200 μl PBS supplemented with 0.1% BSA (PBSF) in 96-well plates (Greiner), spun down for 4 min at 2500 g and supernatant was removed by vacuum aspiration. Cells were the suspended in 50 μl PBSF containing 1:200 dilutions of mouse monoclonal anti-HA tag antibody HA.11 clone 16B12 (Covance) or chicken polyclonal anti-cMyc tag antibody (Gallus Immunotechnology) for detection of display levels, and human HIV antibody at various concentration and incubated for 1 h at ambient temperature while shaking at 1000 rpm. Cells were then washed twice with 200 μl PBSF, suspended in 50 μl PBSF containing 1:200 dilutions of polyclonal goat anti-mouse or goat anti-chicken antibody and goat anti-human antibody conjugated to Alexa488 or Alexa647 (Life Sciences) and incubated for 20 min at ambient temperature while shaking. Cells were again washed once, suspended in 200 μl PBSF, respectively, and kept cold for flow-cytometric analyses. Routinely, cells were gated using forward- and side scatters and median fluorescence intensities (MFIs) were determined from 10,000 scatter-gated events that were then gated for display signal. The gate for display level was set using a non-displaying control sample, assuring that non-displaying cells are excluded. MFI values associated with antibody binding were then normalized to display level by division with the MFI associated with expression tag (nMFIs). All analyses were performed using MACSQuantify software (Miltenyi).

### SDS-PAGE and Western blotting analyses

Yeast-secreted and IMAC purified gp120 was denatured for 5 min at 95° C, loaded on a NuPAGE Novex 3–8% Tris-Acetate gel (Life Scienes), and 150 V were applied for 1 h in MOPS buffer (Life Sciences). The gel was stained using GelCode Blue Stain Reagent (Pierce) and analyzed using a Gel Doc XR System (Bio-Rad). For Western blotting analyses of HIV spike protein variants, typically 20 μl of denatured and reduced crude culture supernatant were loaded and the gel was blotted for 1.5 h at 30 V on a 0.45 μm pore-size PVDF membrane (Life Sciences). The membrane was washed twice for 10 min in PBS, blocked for 1 h in PBS supplemented with 5% BSA and 0.1% v/v Tween 20, washed twice for 10 min in PBS supplemented with 0.05% v/v Tween 20 (PBST) and once in PBS. The washed membrane was incubated with 1 ml PBS containing 3% BSA, 0.1% v/v Tween 20 and 5 μl goat polyclonal anti-gp120 antibody HRP conjugate ab53840 (Abcam) for gp120 detection or 1 μl mouse monoclonal anti-His antibody HRP Conjugate 34460 (Qiagen) for His-tag detection while covered by a transparency. The membrane was then washed as before and incubated for 1 min with enhanced chemiluminescence substrate (Pierce) and analyzed using the Gel Doc XR System (Bio-Rad).

### HIV-1 gp140 library preparation

JR-FL gp140 library insert was prepared based on a protocol by Fromant *et al*. [85], aiming for 5 amino acid mutations per gene. A 100 μl reaction containing 230 μM dATP, 200 μM dCTP, 420 μM dGTP, 2.9 mM dTTP, 512 pM pCHA-d-SOSIP template DNA, 500 nM primer epyd-for2 and 500 nM primer epyd-rev2, 5 μg/ml BSA, 3.95 mM MgCl2, 500 μM MnCl2, 5 u Taq DNA polymerase (NEB) in 50 mM KCl and 10 mM Tris-HCl pH 8.7 at 25° C. Thermo-cycling conditions were 94° C for 5 min, followed by 16 cycles of 94° C for 1 min, 65° C for 1 min and 72° C for 4 min, and finally 72° C for 10 min. PCR products were column-purified and 200 to 800 ng were used as template for amplification in 1000 to 4000 μl of total PCR reaction volume using Phusion DNA polymerase and primers epyd-for3 and epyd-rev3. Thermocycling conditions were 98° C for 1 min, followed by 35 cycles of 98° C for 10 s, 60° C for 30 s and 72° C for 1 min, and finally 72° C for 10 min. Library vector was prepared by cleaving 250 μg of pCHA-SOSIP-JRFL-gp140-design5 with 400 u of BamHI and NheI, respectively, in 1 ml final volume for 8 h at 37° C, followed by the addition of another 400 u of each enzyme, DTT to 1 mM and incubation over night at 37° C. The reaction was heat-inactivated for 20 min at 65° C. Vector was gel-extracted using a DNA Extraction Maxi Kit (Geneaid). Both insert and vector preparations were ethanol-precipitated, washed and concentrated with Pellet Paint Co-Precipitant (Millipore) and suspended in a final volume of approximately 200 μl (insert) and 80 μl (vector). A total of 1200 μl electrocompetent *S*. *cerevisiae* EBY100 were prepared essentially as described previously[84], mixed with all insert and vector, split in eight 2 mm cuvettes and transformed at 1.2 kV and 25 uF using a Gene Pulser Xcell (Bio-Rad). Transformed cells were grown in typically 500 ml SDCAA medium and passaged at least once prior to induction and FACS. Library size was determined by plating dilutions of the transformation on SDCAA plates and counting colonies.

### DNA shuffling

DNA encoding the enriched and designed gp140 variants was shuffled based on a protocol by Stemmer[86]. Briefly, 2.5 μg of gp140 PCR products were incubated with 1 u DNAseI in 50 μl for exactly 2 min at 24° C, respectively, followed by immediate heat-inactivation at 75° C for 10 min. Reactions were separated on a 2% agarose gel and fragments between 25 bp and 1 kb were gel-extracted. Approximately 80 ng of digested, selected gp140 pool was assembled with 80 ng of digested, initial d-SOSIP in 20 μl reactions using Phusion DNA polymerase and 30 s at 98° C followed by 40 cycles of 10 s at 98° C, 30 s at 55° C, 45 s at 72° C and finally 10 min at 72° C. Exactly 2 μl of assembly products were amplified in 50 μl reactions using primers epyd-for3, epyd-rev3 and phusion DNA polymerase during 1 min at 98° C, 30 cycles of 10 s at 98° C, 30 s at 60° C and 1 min at 72° C and finally 5 min at 72° C. The assembled PCR product was ethanol-precipitated, washed, concentrated and used for library preparation as described in the previous section.

### Library selection

Freshly grown yeast libraries were diluted to an OD600 of 0.2 while oversampling the library size at least 10-fold and grown for at least 6 h at 30° C while shaking at 250 rpm. Cells were sedimented, suspended in SGCAA medium and induced for at least 12 h at 30° C. Induced cells were filtered using a 40 μm mesh (BD biosciences) and typically 2x10^8^ cells were washed 3 times with 1 ml PBSF and incubated for 1 h at ambient temperature with varying concentrations of HIV antibodies (Table 1) and optionally a 1:200 dilution of chicken polyclonal anti-cMyc tag antibody (Gallus Immunotechnology) in 500 μl PBSF. Cells were washed twice with 1 ml PBSF and stained for 20 min on ice with 1:200 dilutions of goat anti-human polyclonal antibody conjugated to Alexa647 (Life Sciences) and optionally goat anti-chicken polyclonal antibody conjugated to Alexa488 (Life Sciences), while shielded from light. Cells were washed twice, split in four aliquots and kept as pellets on ice and shielded from light until sorting. FACS was performed using an iCyt sy3200 cell sorter system (Sony). Briefly, aliquots of typically 4x10^7^ stained yeast cells were suspended in 2 ml PBSF and sorted at around 10,000 events/s using 0.1% to 5% sorting gates in recovery mode during initial and ultra purity mode during later sort cycles. Typical sorting gates for initial and later selection rounds are shown in S2 Fig.. Sorted cells were suspended in SDCAA medium and grown for 12 to 36 h for subsequent sorting rounds or analyses of selected pools.

### Biosensor binding studies

All binding analyses were performed at 25° C using an Octet RED96 System (ForteBio), 1000 rpm shaking speed and PBS containing 0.1% BSA, 0.02% v/v Tween20 as sample buffer. Protein A-coated biosensor tips were loaded for 10 min with 10 μg/ml IgG, dipped in buffer and probed with 500 nM gp120 for 8 min, followed by a dissociation phase of 5 min in buffer. A trace obtained from IgG probed with buffer only was subtracted as reference.

### Molecular Modelling

The gp120 crystal structure used for modeling was taken from 4NCO chain A. The CD4-binding site was determined using PyMol interface residues script on gp120-CD4 co-crystal structure PDB ID: 1GC1 and mapped to the corresponding side chains on 4NCO. Mutations of d-SOSIP picked up during selections were mapped onto the BG505 gp140.664 SOSIP structure *via* sequence alignment. Side chain rotamers with minimal clashes were chosen. Hydrogen bonds were designated at distance of <4 Å and >2 Å. Mutation D180N and those in gp41 were not mapped due to lack of resolution in the crystal structure.

## Supporting Information

S1 Fig Characterization of yeast-secreted gp120. (A) SDS-PAGE analysis of JR-FL gp120 secreted from either *S*. *cerevisiae* strain YVH10 (1) or *P*. *pastoris* strain SuperMan5 (2). (B) Octet biosensor binding analysis of *S*. *cerevisiae* or *P*. *pastoris*-secreted gp120. Maximum binding responses during association phase are plotted in nm for a panel of HIV gp120 antibodies and human IgG1 (hIgG1) as reference. (C) Western blotting detection of *S*. *cerevisiae*-secreted and SOSIP gp140 or d-SOSIP with and without PNGase F-treatment to remove N-linked glycans, the black vertical line separates non-adjacent lanes originating from the same blot.
(TIFF)

S2 Fig FACS gate settings. Representative sort gates used during FACS. 100,000 events were recorded, respectively and selection rounds are annotated. Percentages of gated cells are indicated within the respective gates.
(TIFF)

S3 Fig Sequence clustering analysis. Clones sequenced after selection cycle 3.3 and before DNA shuffling (A) or directly after DNA shuffling (B) were clustered using a Blossum90 matrix. Lengths of the branches correlate with sequence similarity. Sequence clusters observed after C3.3 are denoted.
(TIFF)

S4 Fig Pool binding analysis of different selection tracks. Expression-normalized binding response for selection cycle 3.3 track 1 (grey), 4.3 tracks 1 (black), 2 (green) and 3 (blue) are plotted against logarithmic Ab concentrations for VRC01 and PGV04.
(TIFF)

S5 Fig Sequence alignment of lead candidates. Amino acid sequence of d-SOSIP. Residues mutated in clones 3.3.1, 4.3.B01 (B01) and 4.3.D01 (D01) are annotated. The designed Kex2 site is underlined and the designed fusion peptide written in bold letters. The lysine to glutamine mutation to remove an internal, predicted Kex2 site is underlined and bolded.
(TIFF)

S6 Fig d-SOSIP nucleotide sequence. The nucleotide sequence of d-SOSIP gp140 compatible with the primers used in this study is shown.
(TIFF)

S7 Fig Binding analysis of yeast-secreted, soluble gp120 and gp140. Yeast-secreted and purified JR-FL g120 (black) and 4.3.D01 gp140 (grey) were probed for binding to HIV mAb NIH45–46 G54W. The BioLayer Interferometry (BLI) binding response is plotted in nm.
(TIFF)

S1 Table Primers utilized in gene and library construction. (DOCX)

S2 Table Raw data summary of Fig. 2D. (DOCX)
