# Supplementary figures and images for: Directed Evolution of a Yeast-Displayed HIV-1 SOSIP gp140 Spike Protein toward Improved Expression and Affinity for Conformational Antibodies
Source: PLoS One. 2015 Feb 17;10(2):e0117227. doi: 10.1371/journal.pone.0117227 (PMC4331506; doi:10.1371/journal.pone.0117227)

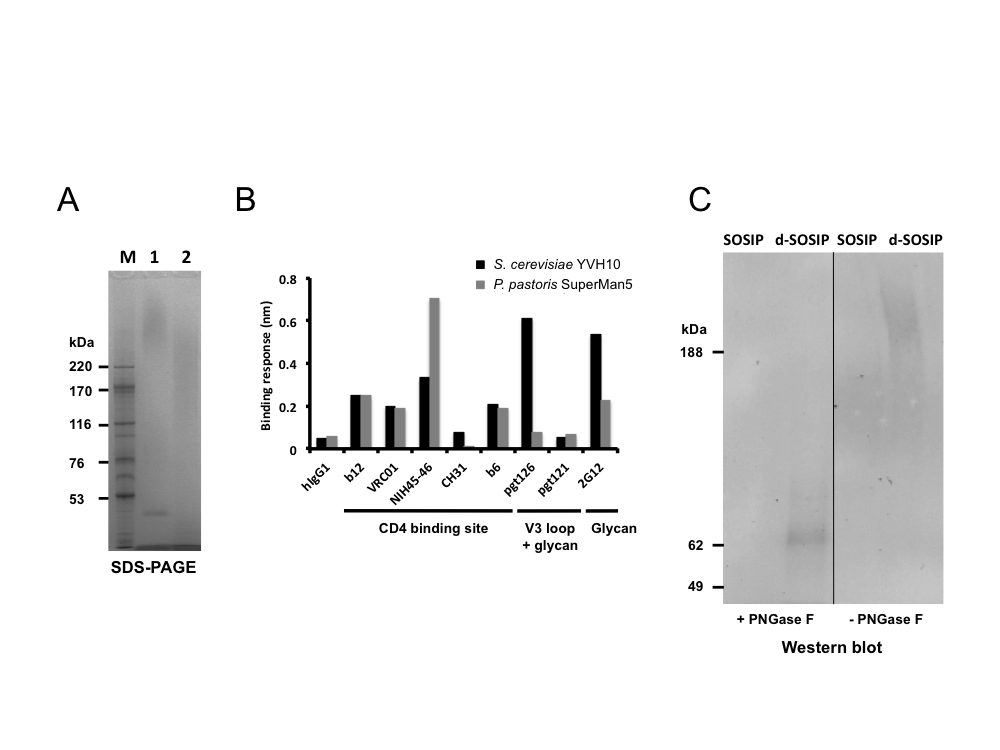

Supplement: S1 Fig — (A) SDS-PAGE analysis of JR-FL gp120 secreted from either S. cerevisiae strain YVH10 (1) or P. pastoris strain SuperMan5 (2). (B) Octet biosensor binding analysis of S. cerevisiae or P. pastoris-secreted gp120. Maximum binding responses during association phase are plotted in nm for a panel of HIV gp120 antibodies and human IgG1 (hIgG1) as reference. (C) Western blotting detection of S. cerevisiae-secreted and SOSIP gp140 or d-SOSIP with and without PNGase F-treatment to remove N-linked glycans, the black vertical line separates non-adjacent lanes originating from the same blot. (TIFF) [file pone.0117227.s001.tiff]

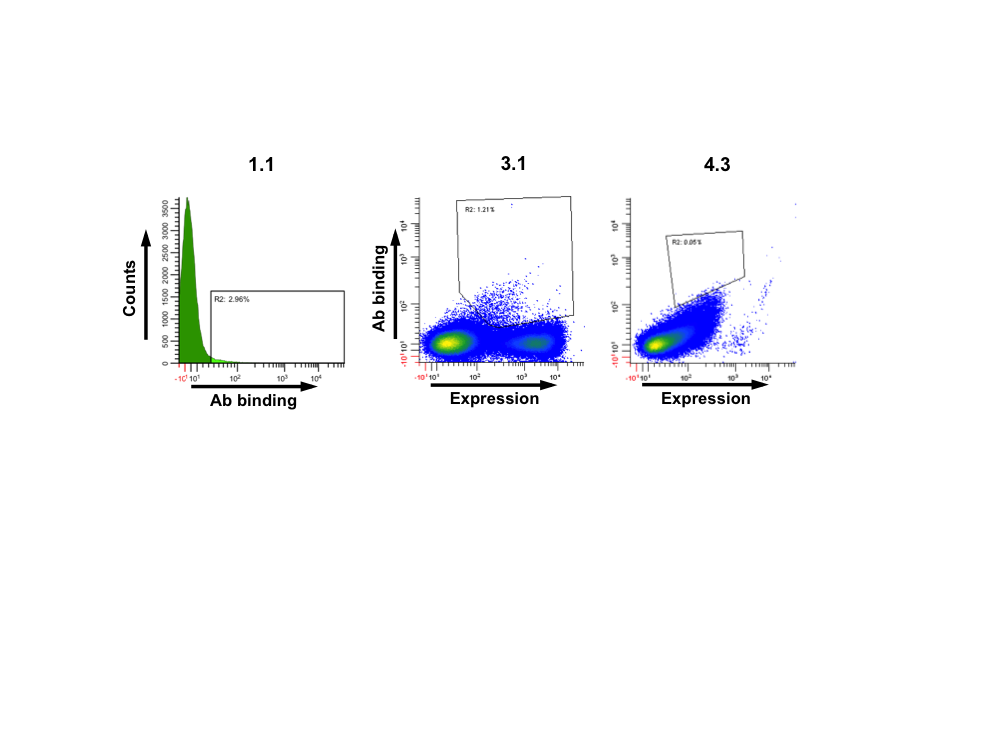

Supplement: S2 Fig — Representative sort gates used during FACS. 100,000 events were recorded, respectively and selection rounds are annotated. Percentages of gated cells are indicated within the respective gates. (TIFF) [file pone.0117227.s002.tiff]

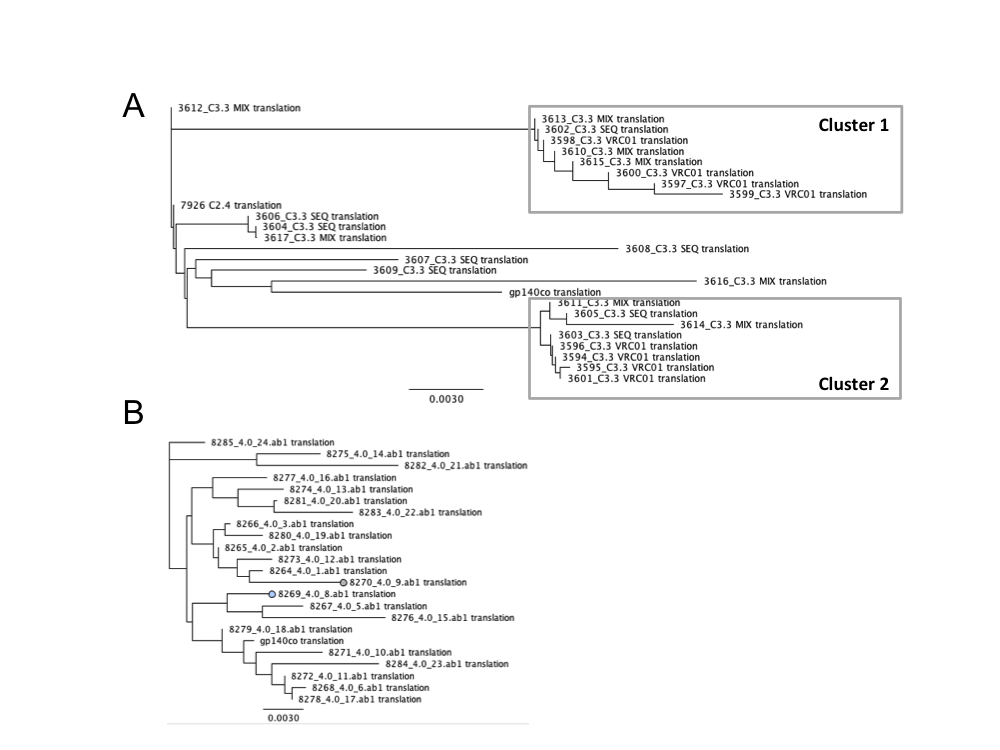

Supplement: S3 Fig — Clones sequenced after selection cycle 3.3 and before DNA shuffling (A) or directly after DNA shuffling (B) were clustered using a Blossum90 matrix. Lengths of the branches correlate with sequence similarity. Sequence clusters observed after C3.3 are denoted. (TIFF) [file pone.0117227.s003.tiff]

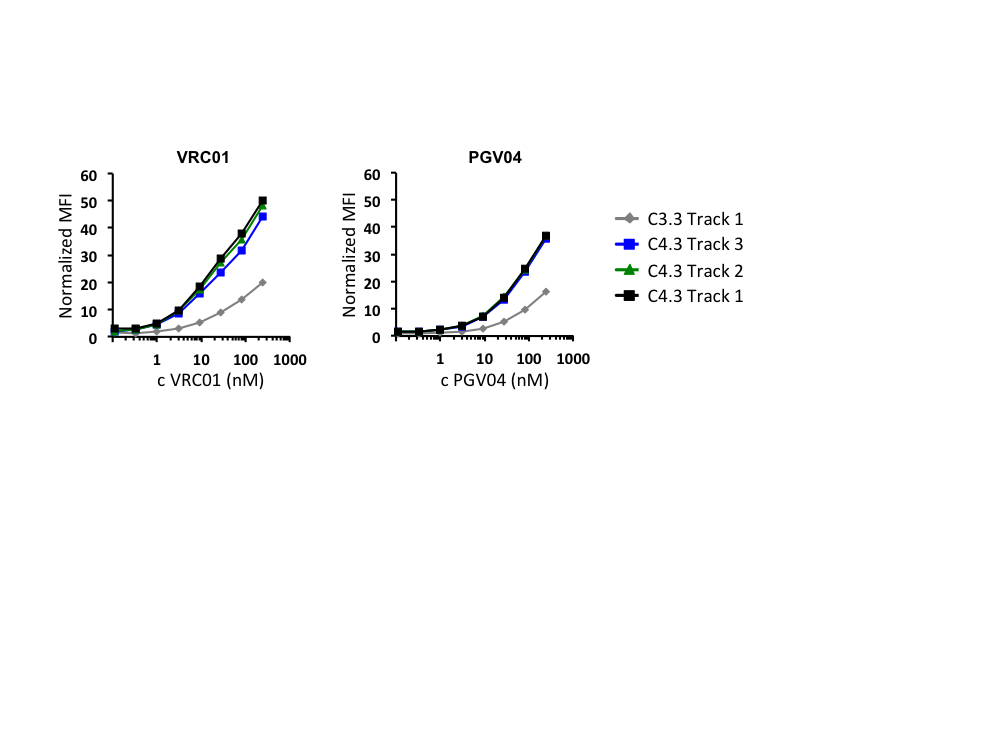

Supplement: S4 Fig — Expression-normalized binding response for selection cycle 3.3 track 1 (grey), 4.3 tracks 1 (black), 2 (green) and 3 (blue) are plotted against logarithmic Ab concentrations for VRC01 and PGV04. (TIFF) [file pone.0117227.s004.tiff]

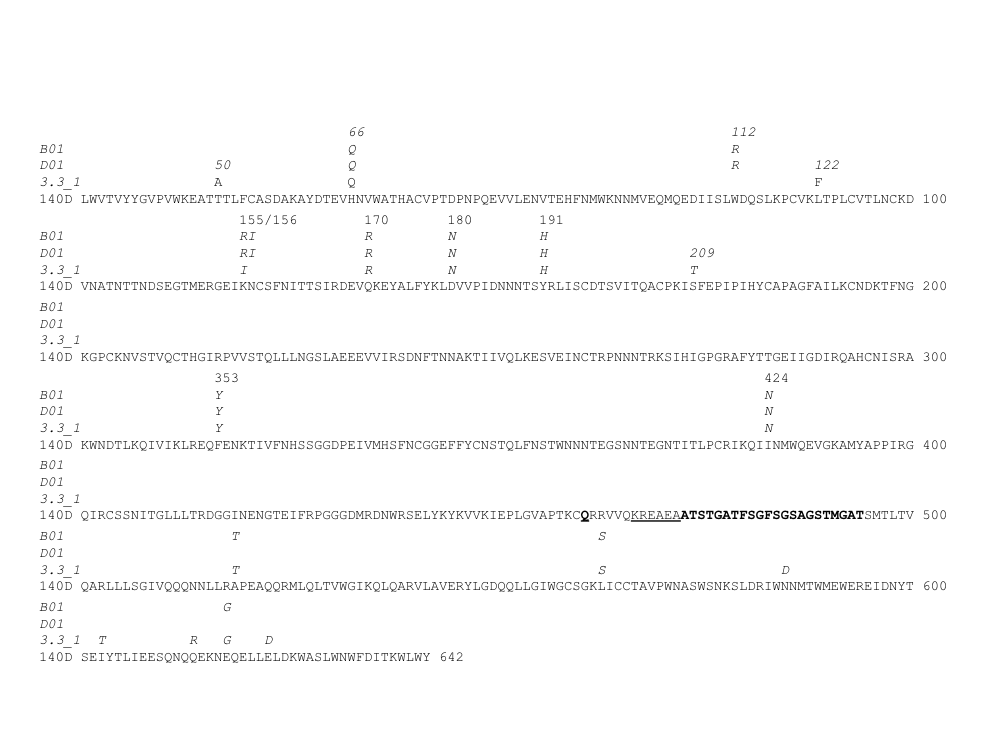

Supplement: S5 Fig — Amino acid sequence of d-SOSIP. Residues mutated in clones 3.3.1, 4.3.B01 (B01) and 4.3.D01 (D01) are annotated. The designed Kex2 site is underlined and the designed fusion peptide written in bold letters. The lysine to glutamine mutation to remove an internal, predicted Kex2 site is underlined and bolded. (TIFF) [file pone.0117227.s005.tiff]

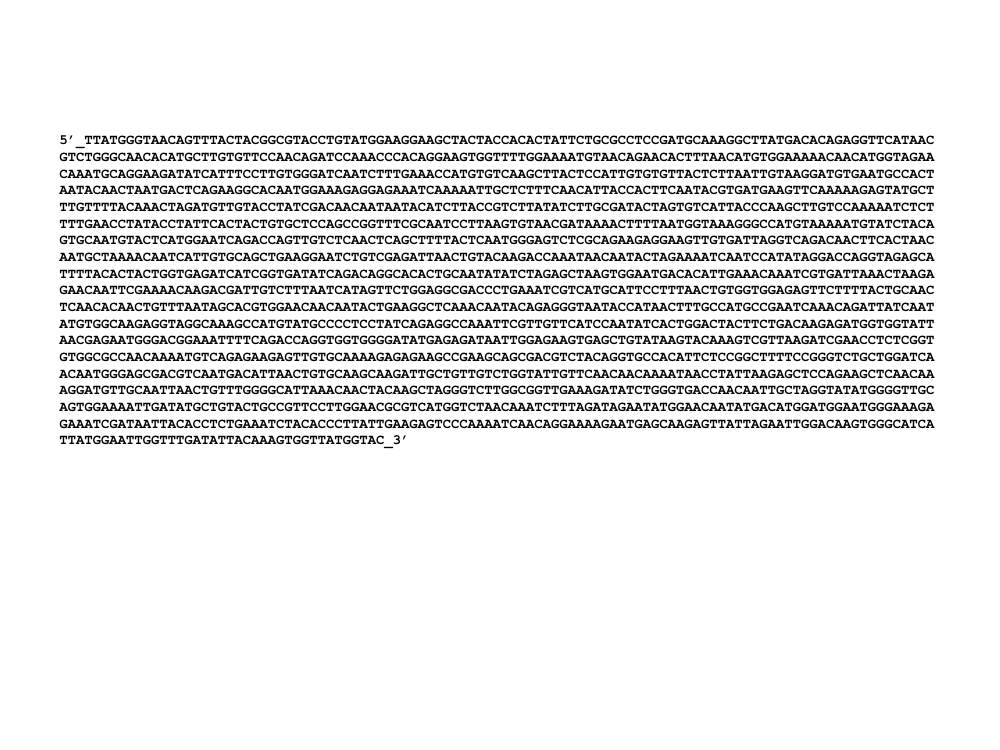

Supplement: S6 Fig — The nucleotide sequence of d-SOSIP gp140 compatible with the primers used in this study is shown. (TIFF) [file pone.0117227.s006.tiff]

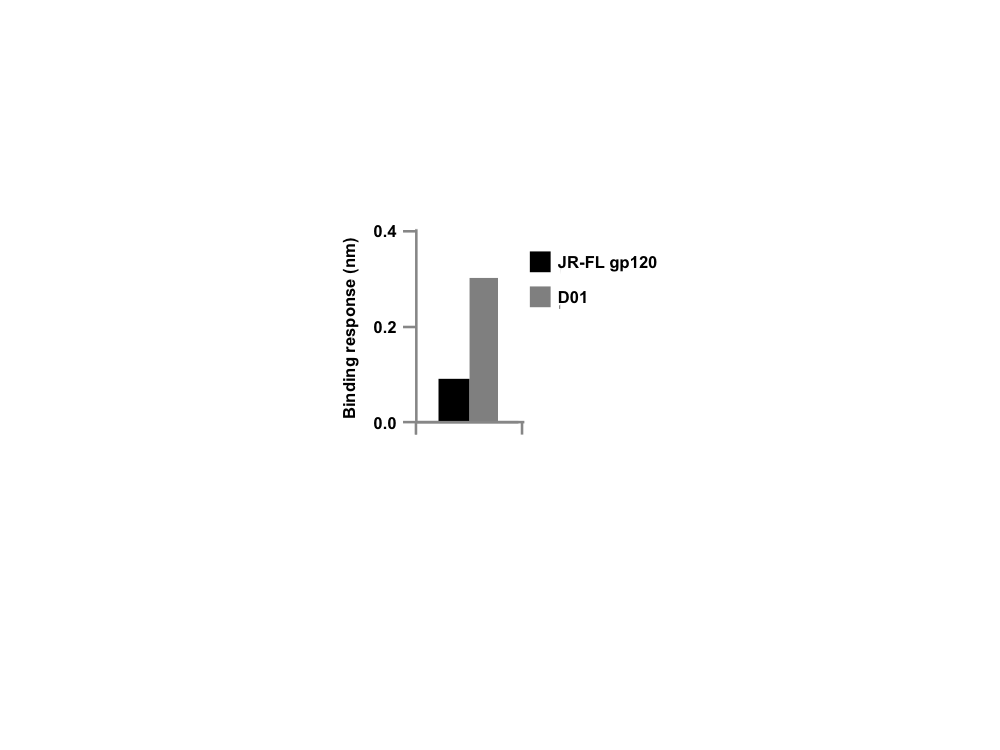

Supplement: S7 Fig — Yeast-secreted and purified JR-FL g120 (black) and 4.3.D01 gp140 (grey) were probed for binding to HIV mAb NIH45–46 G54W. The BioLayer Interferometry (BLI) binding response is plotted in nm. (TIFF) [file pone.0117227.s007.tiff]

**Supporting Table S2: Raw data summary of figure 2D.**


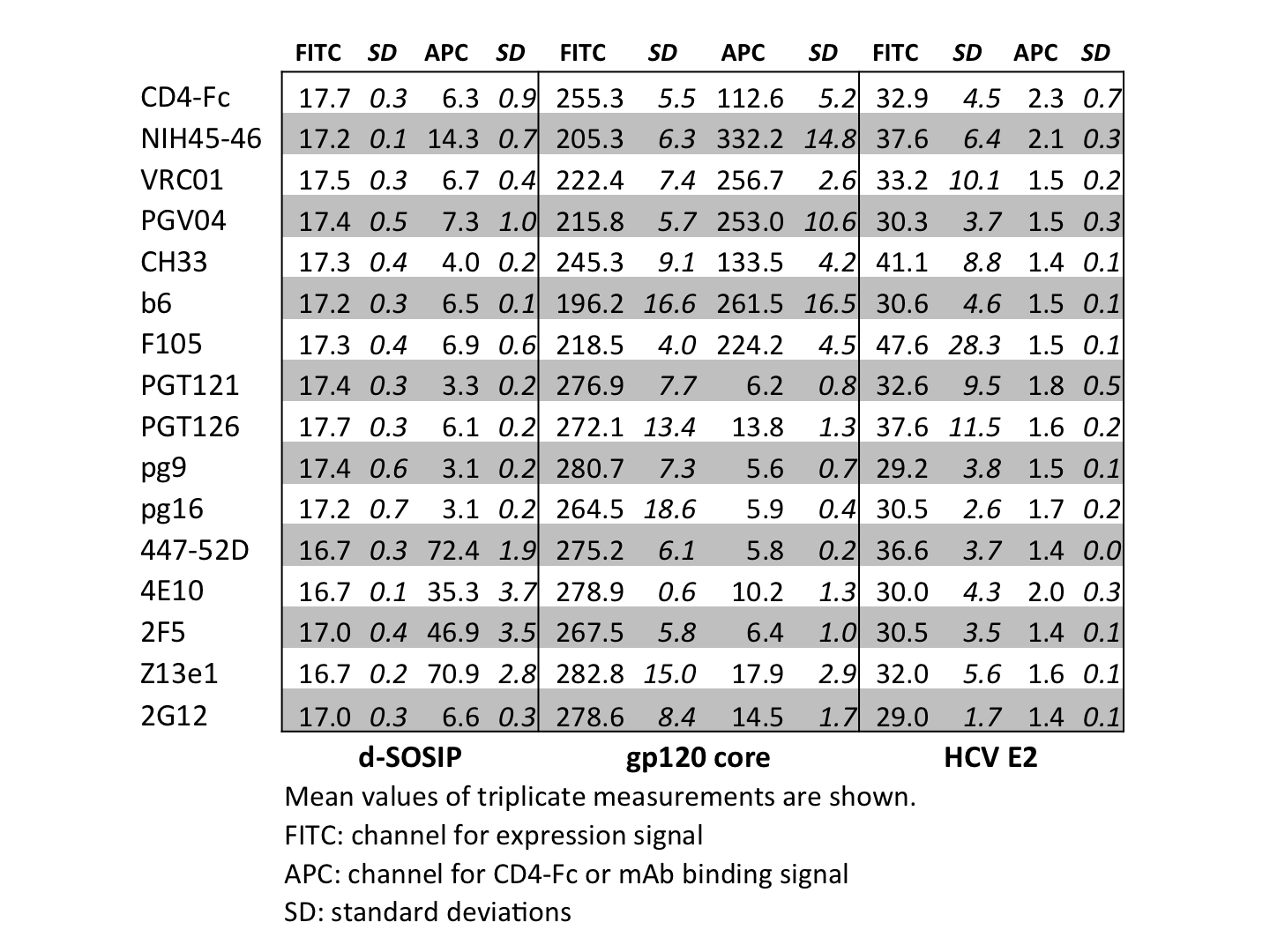

Supplement: S2 Table — (DOCX) [file pone.0117227.s009.docx]
