# Supplementary material for: Directed Evolution of a Yeast-Displayed HIV-1 SOSIP gp140 Spike Protein toward Improved Expression and Affinity for Conformational Antibodies
Source: PLoS One. 2015 Feb 17;10(2):e0117227. doi: 10.1371/journal.pone.0117227 (PMC4331506; doi:10.1371/journal.pone.0117227)
Supplement: S1 Table — (DOCX) [file pone.0117227.s008.docx]

**Supporting Table S1: Primers utilized in gene and library construction.**
